# Missed Coronary Artery Dissection Post-Blunt Chest Trauma

**DOI:** 10.7759/cureus.47630

**Published:** 2023-10-25

**Authors:** Vlad I Delia, Diamantakis Emmanouil, Kotsaki A Theodora, Kontogianni Dimitra, Kotsakis Athanasios

**Affiliations:** 1 2nd Department of Cardiology, General Hospital of Nikea, Piraeus, GRC; 2 Physiology, Medical School, University of Crete, Crete, GRC

**Keywords:** intravascular imaging, optical coherence tomography (oct), coronary artery angiography, coronary artery dissection (cad), blunt trauma chest

## Abstract

Cardiac contusion is rarely diagnosed in patients with blunt chest trauma in the emergency department, especially if patients are asymptomatic. We present a case of a 43-year-old man whose diagnosis of left anterior descending artery (LAD) dissection after blunt chest trauma was delayed. The patient presented to the emergency department of a remote district hospital after a motorcycle accident, asymptomatic with a mildly reduced level of consciousness due to a very small subdural hemorrhage. Ten days later, when he developed a syncopal attack due to a massive pulmonary embolism (PE), his first performed electrocardiogram (ECG) showed sinus rhythm with QS waves and slight ST elevation in leads V2-V6. The subsequent coronary angiography showed left anterior descending artery dissection, and the diagnosis was nicely depicted with optical coherence tomography (OCT). A drug-eluting stent was implanted with a good angiographic result. This case highlights the significance of early recognition of traumatic coronary dissection, which should be excluded even in asymptomatic patients with a plain ECG acquisition, for the appropriate management and prevention of unfavorable outcomes.

## Introduction

Cardiac contusion is rarely diagnosed in patients with blunt chest trauma in the emergency department, especially if they are asymptomatic. Despite its low incidence, physicians should be aware of life-threatening complications that can occur, such as coronary artery dissection (CAD), which can lead to myocardial infarction, life-threatening arrhythmia, and even sudden cardiac death [1]. That is why an ECG is necessary in all patients with thoracic trauma, even in asymptomatic patients, since the early diagnosis of CAD can minimize the cardiac damage and mortality rate [2]. We present a case of a 43-year-old man whose diagnosis of left anterior descending artery (LAD) dissection after blunt chest trauma was delayed.

## Case presentation

**Figure 1:**
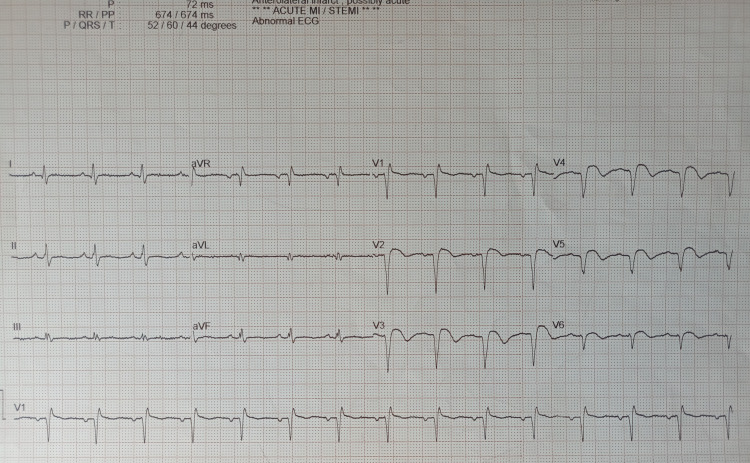
Electrocardiogram demonstrating sinus rhythm with QS waves and slight ST-segment elevations in the anterior leads

**Figure 2:**
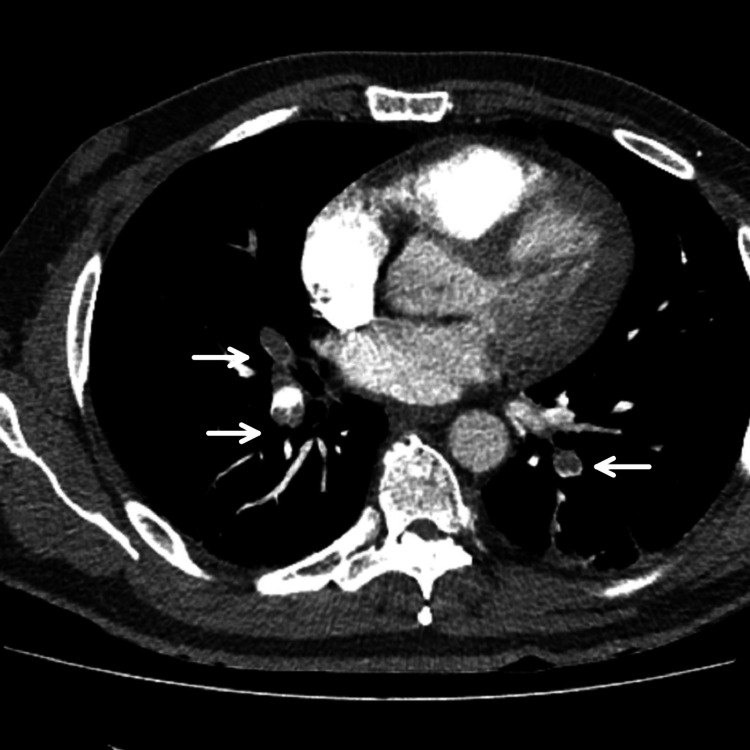
Computed tomography pulmonary angiography (CTPA) documented massive pulmonary embolism with intraluminal filling defects (arrows) of the right and left pulmonary arteries

This is the case of a 43-year-old smoker with a history of pulmonary embolism (PE) 15 years ago, post-surgery, who presented to the emergency department of a remote district hospital after a motorcycle accident. On arrival, the patient was hemodynamically stable. He underwent a full-body computed tomography (CT) scan, showing a very small subdural hemorrhage. There was no pericardial effusion, hemothorax, or pneumothorax. Due to a mildly reduced level of consciousness (a Glasgow Coma Score (GCS) of 13/15), he was admitted to the hospital for observation. He remained asymptomatic and stable until the 10th day when he developed a syncopal attack. The ECG showed sinus rhythm with QS waves and slight ST elevation in leads V2-V6 (Figure 1). He had a CT pulmonary angiogram showing massive PE (Figure 2) and repeated the brain CT with no signs of subdural hemorrhage. He was immediately started on anticoagulation therapy with low-molecular-weight heparin and was transferred to our hospital for further treatment.

**Figure 3:**
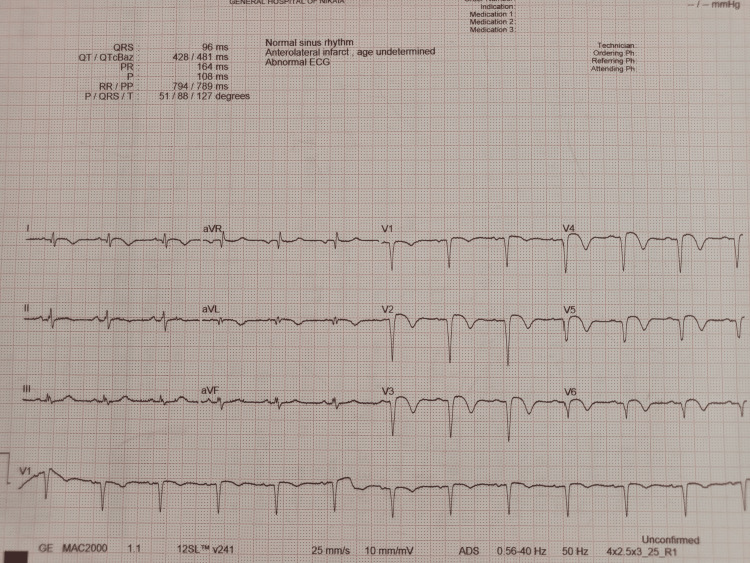
Electrocardiogram demonstrating sinus rhythm with QS waves and slight ST-segment elevations in leads V2-V6 and negative T waves in leads I and aVL

Upon admission to our cardiac department, the patient was asymptomatic. A physical examination showed a blood pressure of 110/70 mmHg, normal saturation, and a pulse rate of 89 beats per minute. Cardiac auscultation was normal, and the lungs were clear. The admission ECG in our hospital showed, apart from QS waves and slight ST elevation in leads V2-V6, inverted T waves in leads I and aVL, which did not exist in the ECG from the referring remote hospital (Figure 3). The chest X-ray was normal. In the patient’s initial blood tests, troponin concentration was 887 ng/l (normal values <72 ng/l), with gradual reduction over the next days (the troponin value on the discharge day was 113,5 ng/l). The initial differential diagnosis included ST-segment elevation, myocardial infarction, and stress-induced cardiomyopathy. Further laboratory investigations with a full blood count showed an elevated white blood cell count of 11750/mL, while common blood chemistry and lipid profile were all within normal values. The transthoracic echocardiogram showed severe hypokinesia in the anterior and apical septal left ventricular wall, with an estimated ejection fraction of 35%. Due to the fact that the patient has been diagnosed with PE and acute coronary syndrome at the same time, we proceeded to check his coagulation status, which did not reveal any abnormalities.

**Figure 4:**
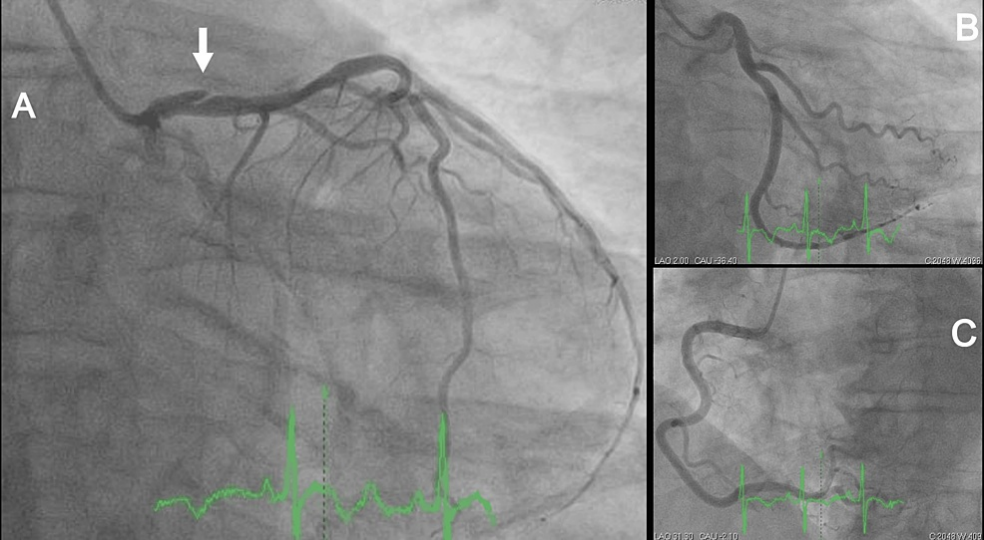
A. Left anterior descending artery with proximal coronary artery dissection (arrow) B. Left circumflex artery C. Right coronary artery

**Figure 5:**
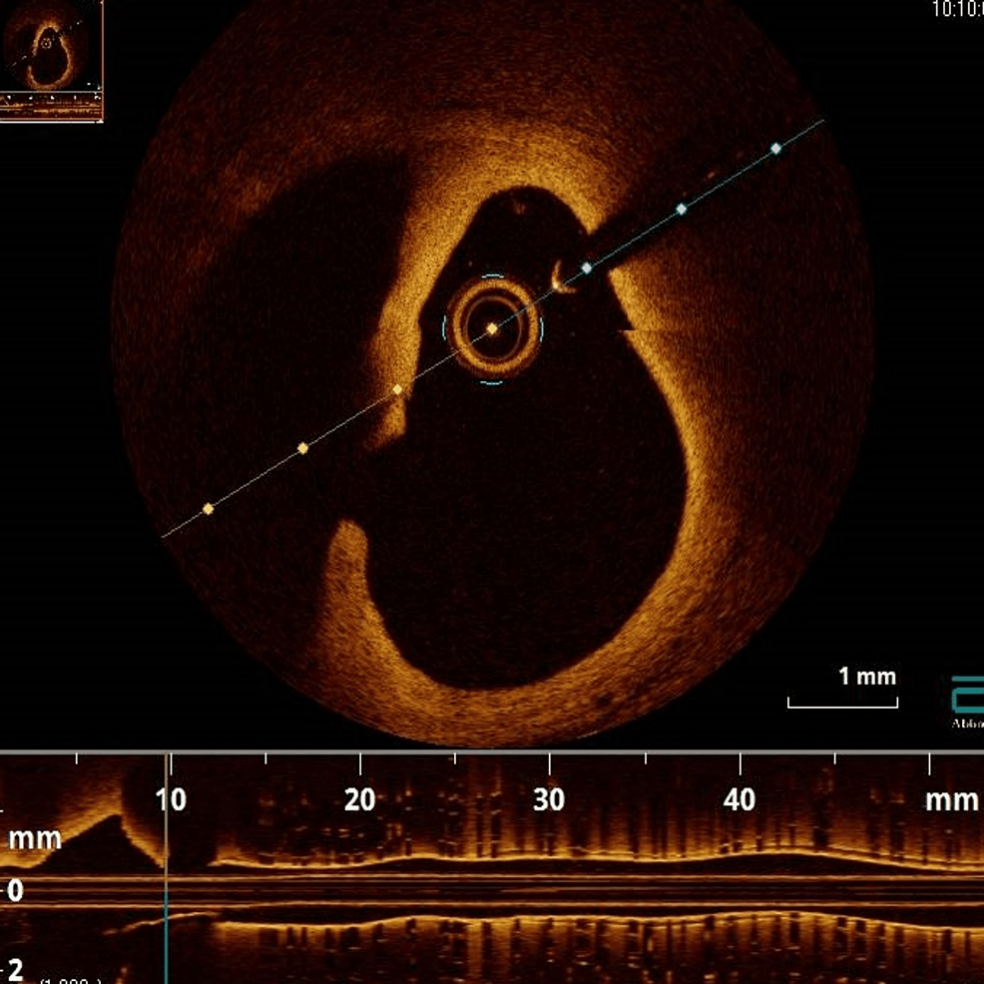
Optical coherence tomography showing the dissection flap and a very large intramural hematoma in the proximal LAD LAD: left anterior descending artery

The patient was led to the cath lab, where coronary angiography showed separate ostia of the LAD and left circumflex artery (LCx), with a long dissection flap in the proximal LAD (Figure 4A). The LCx (Figure 4B) and right coronary artery (RCA) were angiographically normal (Figure 4C). We decided to use optical coherence tomography (OCT) just to confirm the diagnosis. This method of intravascular imaging clearly showed the dissection flap and a very large intramural hematoma in the proximal LAD (Figure 5, Video 1).

**Video 1:** OCT pull back showing the dissection and haematoma in the proximal LAD OCT: optical coherence tomography; LAD: left anterior descending artery

**Figure 6:**
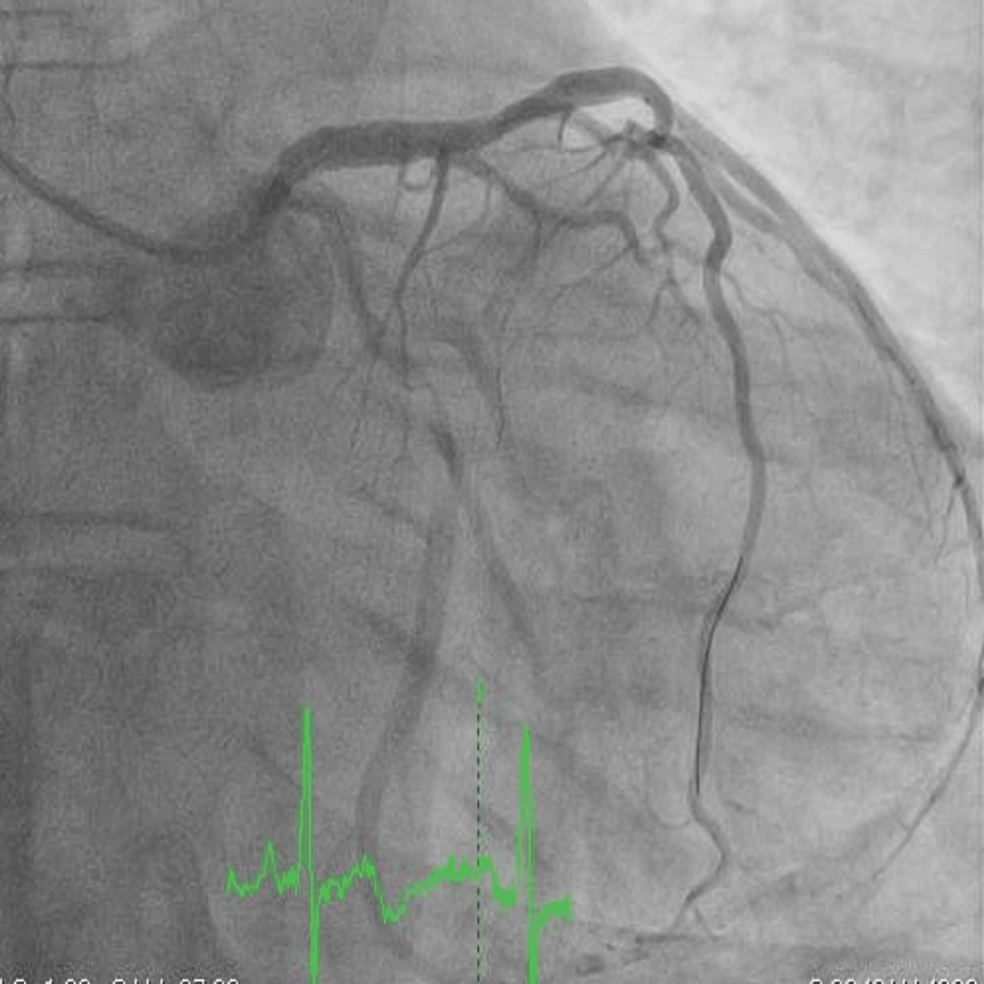
Proximal left anterior descending artery after percutaneous coronary intervention with stent implantation

**Figure 7:**
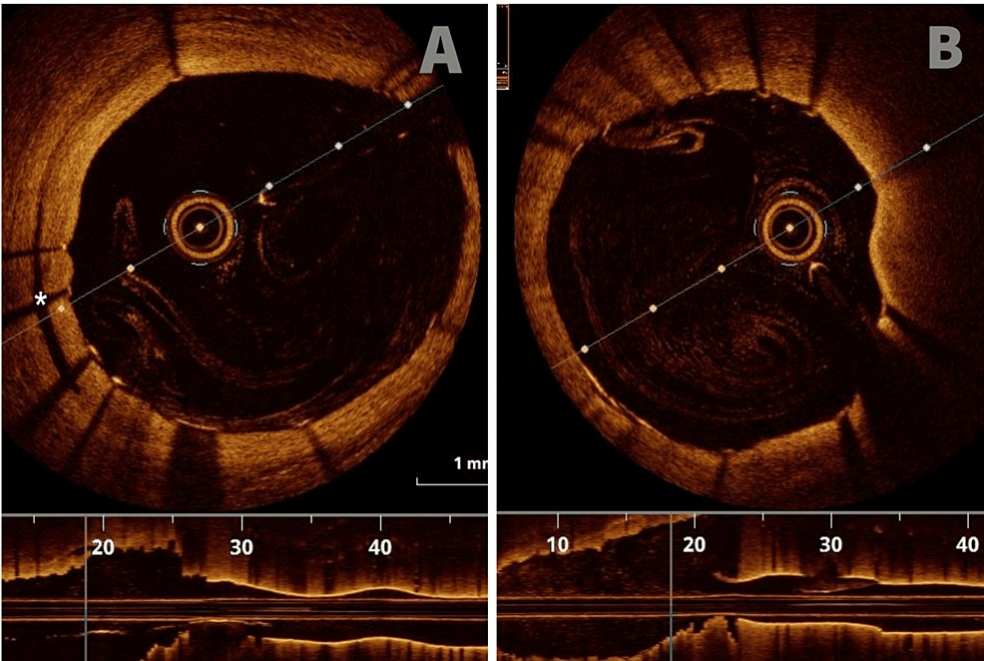
A. LAD after stent implantation, with a residual intramural hematoma (*). B. Final angiographic result after stent post-dilatation LAD: left anterior descending artery

The final diagnosis was a traumatic coronary artery dissection. Due to the fact that the left main was nonexistent and the dissection involved only the proximal LAD, we decided to perform percutaneous coronary intervention (PCI) with the guidance of OCT, despite the absence of any symptoms. After the guidewire was passed to the distal LAD, OCT was used again to make sure the guidewire was in the true lumen. A drug-eluting stent (DES) (4x16mm) was directly implanted, and a post-dilatation with a non-compliant (NC) balloon 4.5x12mm was performed (Figure 6). After the stent implantation, the OCT image showed a residual intramural hematoma (Figure 7A). Repeat OCT after the post-dilatation with the NC balloon revealed a satisfactory final angiographic result with good stent deployment and apposition (Figure 7B). The patient received medical treatment with clopidogrel, apixaban (for his massive PE), an ACE inhibitor, a beta blocker, and statin. Five days post-intervention, he was discharged home on the above medication.

In the six-month follow-up visit, the patient has remained asymptomatic and hemodynamically stable, with no change in his left ventricular ejection fraction.

## Discussion

Blunt chest trauma can cause severe thoracic injuries like pneumothorax or hemothorax. Can also cause severe cardiac injuries, including the myocardium, pericardium, large thoracic vessels, and coronary arteries, most commonly artery dissection [3].

Coronary artery dissection has a very low incidence, and those due to blunt chest trauma are even less common (approximately 0.1%) [4]. A traumatic coronary dissection is more commonly observed in the LAD (76%), due to his anterior position, than in the RCA and LCx (12% and 6%, respectively) [5]. The symptoms vary, from patients who are completely asymptomatic to patients with acute coronary syndrome, but they can present even with sudden death [3]. That is why early recognition is critical for survival. Cardiac chest pain is often difficult to diagnose in trauma due to distracting injuries and chest wall contusions. Current Eastern Association for the Surgery of Trauma (EAST) guidelines propose that an ECG and cardiac injury markers should be obtained in all patients in whom blunt cardiac injury is suspected [6]. There are no guidelines suggesting screening every patient with an early ECG after blunt chest trauma. Thus, obtaining an ECG upon presentation can be wrongly omitted in asymptomatic patients with blunt chest trauma. Our patient was completely asymptomatic at the presentation, which is why he had his first ECG ten days later. All patients presenting with blunt chest trauma, apart from assessment for hemodynamic stability, should have a screening ECG to assess for ischemic changes or arrhythmias.

Echocardiography is the second most valuable tool that can be used in the emergency department for all patients with blunt chest trauma. Echocardiography provides structural and functional assessments of the heart and helps us rule out cardiac injuries, including valvular dysfunction, septal or free wall rupture, cardiac tamponade, and ventricular wall hypokinesia or akinesia [7].

All those patients with ischemic changes on the ECG should undergo coronary angiography examination, which allows visualization of the dissection flap [8]. OCT is an intravascular imaging tool that can confirm and provide detailed characteristics of the dissection flap, guide treatment, and verify the results of interventions [9,10]. The management of coronary artery dissection is patient-dependent and varies from conservative treatment to bypass grafting [8]. However, thrombolysis has to be avoided, especially in patients with injuries that can cause massive bleeding. The preferred therapy for those patients with LAD dissection is PCI with stent implantation. We applied this treatment to our patient, with a good final result.

## Conclusions

This case highlights the significance of early recognition of traumatic coronary dissection due to any type of vehicle collision, even in asymptomatic patients, for the appropriate management and prevention of unfavorable outcomes. Further research is required on the topic of traumatic coronary dissection in order to establish an early, correct diagnosis and treatment, including long-term outcomes.
